# Retraction: Fructose sensitizes pancreatic beta cells to TNFα-induced necroptosis (doi: 10.1242/bio.014712)

**DOI:** 10.1242/bio.014712

**Published:** 2015-10-12

**Authors:** Nataly Shulga, John G. Pastorino

This Research Article has been retracted at the request of the corresponding author, John G. Pastorino.

This notice updates and replaces a recent Expression of Concern, published on 21 January 2016 (see Supplementary information).

Biology Open was alerted to potential blot duplication and reuse in this article. These concerns were relayed to Dr Pastorino, the corresponding author, who responded with an explanation and original data. Following review of these data, we felt unable to resolve this matter at a distance, so contacted the authors’ institution (Rowan University) and requested that they investigate further.

Following their assessment, Rowan University required that Dr Pastorino retract this paper. Dr Pastorino also entered a Voluntary Exclusion Agreement with The Office of Research Integrity (ORI); the agreement can be found here: http://ori.hhs.gov/content/case-summary-pastorino-john-g.

ORI found that Dr Pastorino intentionally falsified and/or fabricated data, and specifically, that he “…duplicated images, or trimmed and/or manipulated blot images from unrelated sources to obscure their origin, and relabelled them to represent different experimental results in:

 • Figures 3A and 6B in Biol. Open 2015” (doi: 10.1242/bio.014712).

The PDF of the retracted article is available as Supplementary information.

Supplementary Material

Retracted article.

