# Supplementary material for: Retraction: Fructose sensitizes pancreatic beta cells to TNFα-induced necroptosis (doi: 10.1242/bio.014712)
Source: Biol Open. 2015 Oct 12;5(7):1010. doi: 10.1242/bio.014712 (PMC4958267; doi:10.1242/bio.014712)
Supplement: Retracted article. [file BIO014712supp.pdf]

Expression of Concern: Fructose sensitizes pancreatic beta cells to TNFα-induced necroptosis by Nataly Shulga and John G. Pastorino. *Biol. Open* doi: 10.1242/bio.014712

Rachel Hackett[1] and Jordan W. Raff[2]
[1]Managing Editor
[2]Editor in Chief (bio@biologists.com)

Rachel Hackett is Managing Editor of Biology Open, and Jordan Raff is Editor-in-Chief of Biology Open and Professor at the Sir William Dunn School of Pathology, University of Oxford.

Concerns have been raised about the scientific validity of article "Fructose sensitizes pancreatic beta cells to TNFα-induced necroptosis" by Nataly Shulga and John G. Pastorino (doi: 10.1242/bio.014712), which is currently posted online as an Advance Article.

BiO takes these matters very seriously for the sake of the scientific record. These concerns have been relayed to the corresponding author, who has responded with an explanation and original data. Following review of these data, we felt unable to resolve this matter, and have contacted the authors' institution and requested that they conduct further investigation.

BiO is posting this Publisher's Note for information purposes only. It is solely intended to alert readers to the situation while the investigation takes place, and is not a statement regarding the validity of the data. We will provide additional information when it becomes available.

This course of action follows the advice set out by COPE (Committee on Publication Ethics) and the International Committee of Medical Journal Editors (ICMJE), of which BiO is a member.

Biology Open • Advance article

© 2016. Published by The Company of Biologists Ltd.
This is an Open Access article distributed under the terms of the Creative Commons Attribution License (http://creativecommons.org/licenses/by/3.0), which permits unrestricted use, distribution and reproduction in any medium provided that the original work is properly attributed.

© 2015. Published by The Company of Biologists Ltd | Biology Open (2015) 0, 1-11 doi:10.1242/bio.014712

RESEARCH ARTICLE

Fructose sensitizes pancreatic beta cells to TNFα-induced necroptosis

Nataly Shulga and John G. Pastorino*

RETRACTED

ABSTRACT

The past half-century has witnessed a dramatic increase in the incidence of obesity and diabetes (Jacobson, 2004; Krilanovich, 2004). Both of these occurrences have been accompanied by an increase in the consumption of fructose. Unlike glucose, the metabolism of fructose is not subject to negative feed-back inhibition and can impose stress on intracellular energy stores (Ishimoto et al., 2012; Lanaspa et al., 2014, 2012). In the present study we identify the ability of fructose to increase the sensitivity of pancreatic beta cells to TNFα induced cytotoxicity. Exposure of pancreatic beta cells to fructose induced fructokinase and glut-5 expression, two proteins critical for the metabolism of fructose. Importantly, the increased metabolism of fructose by beta cells was accompanied by an increase in the expression of mitoNEET. MitoNEET is a 2Fe-2S cluster binding protein localized to the outer mitochondrial membrane (Wiley et al., 2007a). The increased expression of mitoNEET mediated an enhanced sensitivity of the pancreatic beta cells to TNFα induced cytotoxicity that was prevented by suppression of mitoNEET expression or pharmacological inhibition of its ability to release its 2Fe-2S cluster.

KEY WORDS: Fructose, Necroptosis, Beta cell, TNFα, MitoNEET

INTRODUCTION

Type I diabetes is partly due to pancreatic beta cell death mediated by cytokines. Type II diabetes is also characterized by a gradual loss of beta cell mass (Cnop et al., 2005; Mulder and Ling, 2009; Muoio and Newgard, 2008). Fructose use as a sweetener has grown in the past half century and is hypothesized to contribute to the burgeoning epidemic of diabetes in the developed world; however little is known regarding its effects on beta cell viability (Dekker et al., 2010; Gore et al., 2011; Kmietowicz, 2012). The increased use of fructose has been linked to a heightened incidence of Type II diabetes (Gora et al., 2013). In contrast to the effects of fructose on the liver, the consequences of fructose exposure on pancreatic beta cells are not well characterized. Most fructose is metabolized in the liver by fructokinase. However, some fructose does enter circulation, with fructose blood levels varying within between 5 µM and 1.9 mM following ingestion, suggesting that other cell types can be exposed to significant levels of fructose.

Accumulating evidence suggests that the gradual loss of beta cell viability in type II diabetes is due to an infiltration of activated immune cells, such as macrophages (Ehses et al., 2008; Jordan et al., 2013). The metabolism of fructose is not subject to negative feedback inhibition and can therefore alter the metabolic state of a cell, making it more susceptible to activation. It has been demonstrated that fructose can increase the expression of Toll-like receptors 1-9 in F4/80 positive macrophages (Wagnerberger et al., 2012). We have demonstrated that fructose exposure can lead to an increase in the activation of macrophages due to an elevation in the binding of hexokinase II to mitochondria (Shulga and Pastorino, 2014a). In turn, activated macrophages secrete chemokines and cytokines that can cause damage and loss of beta cell viability. In particular, beta cells express TNFα receptors and are capable of undergoing TNFα induced cytotoxicity (Stephens et al., 1999).

TNFα signaling is complex and can eventuate in many outcomes. However, in some instances the cell can be primed to undergo TNFα induced cell death. Indeed, we have shown that fructose can bring about an increase in the sensitivity of hepatocytes to TNFα induced cytotoxicity (Shulga and Pastorino, 2014b). This is facilitated by an increase in the expression of mitoNEET. MitoNEET is a 2Fe-2S cluster binding protein that is localized to the outer mitochondrial membrane and mediates the maturation and shuttling of 2Fe-2S clusters (Lin et al., 2007). However, we have found that under certain circumstances, mitoNEET can mediate mitochondrial injury and cell death during exposure to TNFα. In this instance, the form of cell death is necroptotic, originating from the necrosome complex composed of RIPK-1 and RIPK-3. The necrosome phosphorylates Stat-3, which induces its interaction with Grim-19, a component of complex I of the mitochondrial respiratory chain that also resides partially in the cytosol and nucleus (Shulga and Pastorino, 2012). The resulting Stat-3-Grim-19 complex translocates to the outer mitochondrial membrane where it binds to mitoNEET, inducing a rapid and massive release of its bound 2Fe-2S complex that is quickly degraded. The resulting free iron is taken up by the mitochondria via the calcium uniporter, where it brings about increased reactive oxygen species (ROS) production and mitochondria permeability transition.

The present study demonstrates that exposure to fructose resulted in a priming of cultured pancreatic beta cells to TNFα induced cytotoxicity. Moreover, beta cells in pancreatic islets isolated from mice fed a fructose containing diet also exhibited a dramatic increase in sensitivity to TNFα induced cell killing. Fructose exposure induced the expression of mitoNEET, in addition to fructokinase and glut-5 in pancreatic beta cells. Induction of mitoNEET, fructokinase or glut-5 expression was necessary for the sensitizing effect, as their suppression prevented the enhanced susceptibility to TNFα cytotoxicity. The data indicate that the induction of TNFα induced necroptosis in beta cells brought about by fructose exposure, is partly caused by the TNFα induced binding of a Stat3-Grim-19 complex to mitoNEET. Additionally,

Department of Molecular Biology, Rowan University School of Osteopathic Medicine, Stratford, NJ 08084, USA.

*Author for correspondence (pastorjg@rowan.edu)

This is an Open Access article distributed under the terms of the Creative Commons Attribution License (http://creativecommons.org/licenses/by/3.0), which permits unrestricted use, distribution and reproduction in any medium provided that the original work is properly attributed.

Received 3 September 2015; Accepted 14 September 2015

Biology Open (2015) 0, 1-11 doi:10.1242/bio.014712

pre-treatment with the anti-diabetic drug, pioglitazone, which binds to and inhibits the ability of mitoNEET to release its 2Fe-2S cluster, prevented the TNFα induced accumulation of mitochondrial iron, ROS formation and cytotoxicity in fructose exposed pancreatic beta cells.

RESULTS

Fructose exposure promotes TNFα induced necroptosis in pancreatic beta cells

To mimic the post-prandial elevation of glucose and fructose that pancreatic beta cells are exposed to, the cultured beta cells were exposed to glucose and fructose containing spikes for 30 min followed by washout with PBS, and then returned to basal RPMI media. INS-1E cells were exposed to three, 30 min spikes of 1 mM fructose and 10 mM glucose separated by 8 h intervals over 24 h. The cells were then treated with TNFα (10 ng/ml) in basal RPMI-1640 media containing 5 mM of glucose (basal condition) for the times indicated. Treatment with TNFα in cells exposed to 3 spikes of 10 mM glucose alone displayed little cytotoxicity as assessed by loss of plasma membrane integrity (PI positive) [Fig. 1A, labeled TNF(+Gluc)]. Exposure to three spikes of 1 mM fructose and 10 mM glucose in and of itself did not bring about any appreciable loss of cell viability [labeled (+Fruct.+Gluc) [illegible] 1A]. By contrast, when the cells were first exposed to th[illegible]ructose [illegible]taining spikes, followed by treatment with TNFα, [illegible]e was a dram[illegible] loss of cell viability, with 60% of the ce[illegible]staining positi[illegible] for propidium iodide at 24 h [labeled TN[illegible](+Fruc[illegible]Gluc)]. Import[illegible]tly, the cell death was prevented by n[illegible]ostatin, an [illegible]bitor of R[illegible]K-1

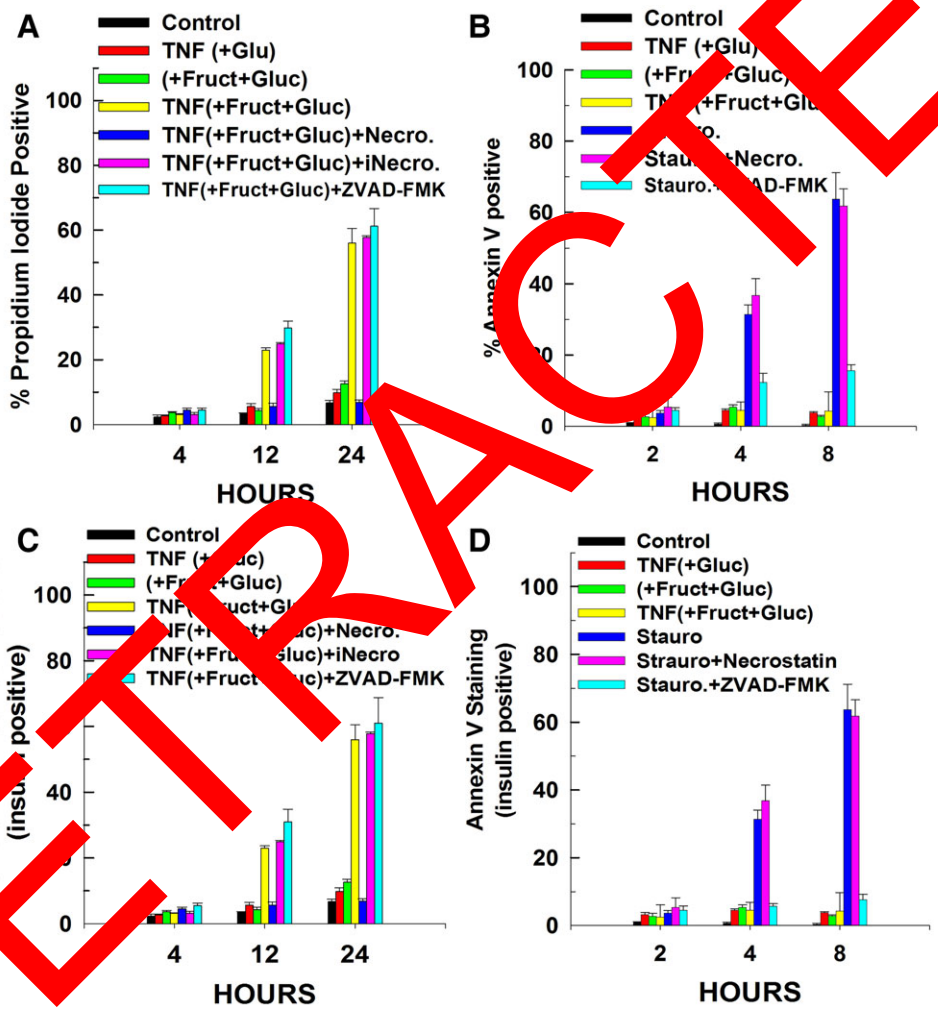

Fig[illegible] Fru[illegible]se expos[illegible]romotes TNFα induced necroptosis in pancreatic beta cells. (A,B) INS-1 cells were plated at 50,000 cells per well in 24 well plates[illegible]ml of basal RPMI media containing 5 mM of glucose. Following 24 h, the cells were exposed to three spikes of 1 mM fructose and 10 mM of glucose. Each spi[illegible]sted for 30 min after which the media was aspirated, the cells washed with PBS and placed back into basal RPMI media containing 5 mM of glucose. Th[illegible]lls were exposed to three spikes in total with an interval of eight hours between spikes. Where indicated, after the last spike, the cells were either un-treated or p[illegible]treated with necrostatin (Necro.), the inactive analog of necrostatin (iNecro.) or the broad spectrum caspase inhibitor, ZVAD-FMK, for 30 min. Following the 30 min pre-treatment, the cells were treated with TNFα at 10 ng/ml for the time periods indicated. Following the treatment, the cells were harvested and viability assessed by propidium iodide uptake or annexin V staining. Values are the means of three independent experiments with the error bars indicating standard deviation (s.d.). (C,D) Mouse pancreatic islets were isolated from mice fed a non-fructose containing diet. The islets were dispersed, cultured for 24 h in basal RPMI media containing 5 mM of glucose. Where indicated, the cells were exposed to three spikes of 1 mM fructose and 10 mM of glucose. Each spike lasted for 30 min, after which the media was aspirated, the islets washed with PBS and placed back into basal RPMI media containing 5 mM of glucose. The cells were exposed to three spikes in total with an interval of eight hours between spikes. After the last fructose-glucose spike, the cells were placed into basal RPMI media, and then untreated or pre-treated with necrostatin, the inactive analog of necrostatin or ZVAD-FMK. Following 30 min, the cells were then treated with TNFα or staurosporin for the times indicated. Cell viability was then determined by the degree of ethidium monoazide or annexin V staining of insulin positive cells. Values are the means of three independent experiments with the error bars indicating s.d.

RETRACTED

Biology Open (2015) 0, 1-11 doi:10.1242/bio.014712

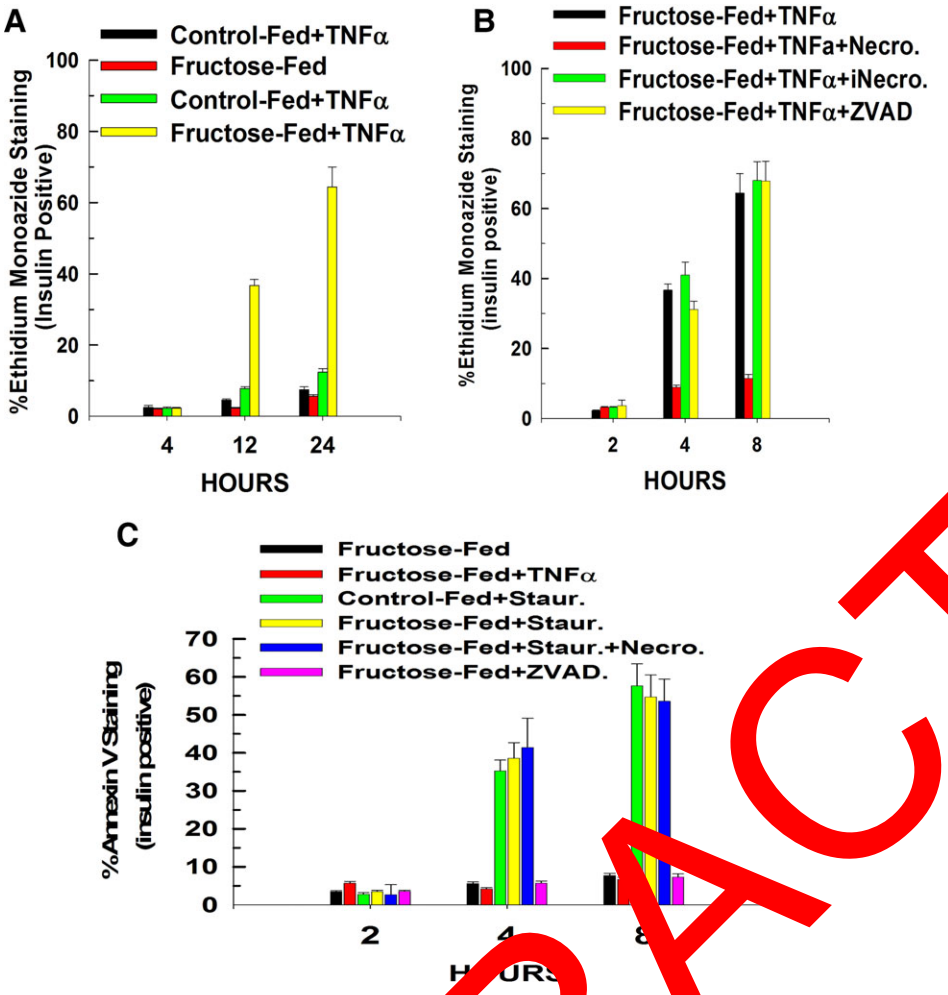

Fig. 2. Pancreatic beta cells of fructose fed mice display increased sensitivity to TNFα induced necroptosis. (A) Mouse pancreatic islets were isolated from mice fed a control or fructose containing diet as outlined in the Materials and Methods. The islets were dispersed, cultured for 24 h in basal RPMI media containing 5 mM of glucose. The islets were then untreated or treated with TNFα over a 24 h time course. Cell viability was then determined by the degree of ethidium mono[illegible] of insulin positive cells. Values [illegible] the means [illegible] ree independent experi[illegible]nts with the error [illegible]s indicating s.d. (B) M[illegible]e pancreatic islets [illegible]ere isolated from [illegible]ice fed [illegible]ctose containin[illegible] diet. The islets [illegible]e dispersed, [illegible]ltured for 2[illegible] in basal R[illegible]n media containin[illegible]mM [illegible]ucose. The[illegible]s were then [illegible]treated o[illegible]eated with ne[illegible]statin, the i[illegible]ctive analog of [illegible]ecrostatin (inec[illegible]or ZV[illegible]D. Following [illegible]0 min, the cells were the[illegible]ated with TN[illegible]for the times indicated. C[illegible]iability [illegible]s then determined by the degree of [illegible]li[illegible]monoazide staining of [illegible]nsulin positive ce[illegible]. Values are the means of [illegible]e independent experiments with the error ba[illegible]dicating s.d. (C) Mouse pancreatic islets were [illegible]ted from mice fed a control or fructose containing diet. The islets were dispersed, cultured for 24 h in basal RPMI media containing 5 mM of glucose. The cells were then untreated or pre-treated with necrostatin or ZVAD. Following 30 min, the cells were un-treated or treated with TNFα or stauropsorine for the times indicated. Cell viability was then determined by the degree of ethidium monoazide staining of insulin positive cells. Values are the means of three independent experiments with the error bars indicating s.d.

(Fig. 1A, +Necro.), with the ina[illegible]e ana[illegible] of necrostatin exhibiting no protective effect (+[illegible]ecro). Signi[illegible]ntly, there was no staining of TNFα and fruct[illegible]xposed cells w[illegible]annexin V at 8 h (Fig. 1B), indicating th[illegible] the c[illegible] death is not a[illegible]ptotic. By contrast, when non-fructose exposed[illegible]ells were treated with staurosporine, the cel[illegible]exhibited annexi[illegible]V staining by 4 h that was not prevented[illegible] necrostatin, but was [illegible]vented by ZVAD-FMK, a broad s[illegible]ctrum cas[illegible]e inhibitor, indicating the mode of cell death in t[illegible] instan[illegible] was apoptotic (Fig. 1B). Moreover, caspase inhibition [illegible]t prevent t[illegible]TNFα induced cytotoxicity seen in f[illegible]e expos[illegible]cells (Fig[illegible]A). Importantly, substitution of fruct[illegible]e with[illegible]s non-m[illegible]bol[illegible]able analog, 3-O-methylfructose, d[illegible] not sens[illegible]e the cell[illegible]o TNFα induced cytotoxicity (not [illegible]wn).

F[illegible]to[illegible] exposure also sensitized primary pancreatic beta cells to TNFα[illegible]uced necroptosis. Pancreatic islets were isolated, the cells dispersed[illegible]d incubated in basal RPMI media containing 5 mM of glucose for[illegible]h. The cells were then exposed to three, 30 min spikes of 1 mM fructose and 10 mM glucose at 8 h intervals. The cells were then treated with TNFα in basal non-fructose containing media for the time periods indicated. Before harvesting the cells, ethidium monoazide or annexin V was added, followed by photo-labeling and washing. The cells were then fixed and assessed for insulin or glucagon content by immunostaining. Importantly, immunostaining with insulin had no effect on annexin V staining as described previously (Medarova et al., 2005). As show in Fig. 1C, in insulin positive islet cells exposed to fructose-glucose spikes and then treated with TNFα, there was a dramatic increase in the number staining positive for ethidium monoazide at 12 and 24 h, indicating a loss of plasma membrane integrity (Fig. 1C, yellow bars). However, the cells did not stain for annexin V after 8 h (Fig. 1D, yellow bars). Importantly, this time point is prior to loss of appreciable plasma membrane integrity, where necrosis would lead to annexin V staining of the inner side of the plasma membrane. As shown in Fig. 1C, pre-treatment with necrostatin prevented the TNFα induced ethidium monoazide staining of the insulin positive islet cells exposed to fructose, but its inactive analog did not. Additionally, the ethidium monoazide staining in fructose and TNFα exposed cells was not prevented by the caspase inhibitor, ZVAD-FMK (Fig. 1C). By contrast, as shown in Fig. 1D, insulin positive islet cells treated with staurosporine displayed annexin V staining by 4 h that was not prevented by necrostatin, but was prevented by the caspase inhibitor. Importantly, islets cells staining positive for glucagon did not show a loss of viability as assessed by ethidium monoazide or annexin V, indicating that the sensitizing effects of fructose are selective for beta cells (not shown).

Pancreatic beta cells were also sensitized to TNFα induced necroptosis by *in vivo* exposure to fructose. Mice were fed a diet that contained 20% of fructose in their drinking water for 2 days. The concentration of fructose in the serum was measured. The serum fructose concentration of the control fed mice was 1.5±0.3 mM. In the fructose fed mice, the initial serum fructose concentration was similar

RETRACTED

Biology Open • Advance article

Biology Open (2015) 0, 1-11 doi:10.1242/bio.014712

to that of controls, at 1.8±0.4 mM. However, following 48 h of fructose feeding, the serum fructose concentration rose to 8.4±0.6 mM. Following 48 h, the islets were isolated, the cells dispersed and cultured for 16 h in fructose-free basal media. It is important to note that in this instance, the islets cells were not exposed to fructose while in culture. The cells were treated with TNFα and assessed for ethidium monoazide, annexin V and insulin or glucagon staining. As show in Fig. 2A, insulin positive islet cells showed no difference in viability between control-fed and fructose-fed mice over 24 h. However, upon treatment with TNFα, there was a marked loss of viability in the insulin positive islet cells isolated from fructose fed mice, with a 36% and 68% loss of viability at 12 and 24 h, respectively. By contrast the insulin positive islet cells isolated from control-fed mice were refractory to TNFα induced cytotoxicity, with only a marginal loss of cell viability at 24 h. As shown in Fig. 2B, pretreatment with necrostatin prevented the TNFα induced cytotoxicity in insulin positive islet cells isolated from fructose fed mice, with the inactive necrostatin analog having no effect. Importantly, the broad spectrum caspase inhibitor, ZVAD, did not display any ability to prevent the loss of cell viability induced by TNFα in cells isolated from fructose-fed mice. These results indicate that the TNFα induced cell killing of fructose exposed cells is necroptotic. In agreement with this assertion, treatment with TNFα did not bring about annexin V staining in insulin positive islet cells isolated from fructose-fed mice (Fig. 2C). By contrast, treatment with the apoptosis inducer, staurosporine, brought about robust annexin V staining in insulin positive islet cells isolated from both control and fructose fed mice (Fig. 2C). Moreover, the annexin V staining induced by staurosporine was prevented by the caspase inhibitor ZVAD, but not by necrostatin (Fig. 2C).

TNFα Induced Necroptosis of fructose exposed beta cells dependent on MitoNEET, fructokinase and glut-5 expression

Fructokinase is expressed at low levels in pancreatic beta cells (Malaisse et al., 1989). However, both glut-5, which is selective for fructose transport, and fructokinase expression have been found to be induced by fructose exposure (Doliba et al., 2008; Koo et al., 2008). Exposure of INS-1E cells to three, 30 min spikes of 10 mM glucose alone did not bring about induction of glut-5, fructokinase or mitoNEET expression (Fig. 3A, lane #1). By contrast, as show in Fig. 3A lane #2, glut-5, fructokinase and mitoNEET expression were induced by exposure to three, 30 min spikes of 1 mM fructose and 10 mM glucose (lane #2). Twenty four hours after fructose exposure, glut-5 and fructokinase expression declined (Fig. 3A, lane #3). By contrast, mitoNEET expression remained elevated even after 24 h following fructose withdrawal (Fig. 3A, lane #3); understanding this may account in part, for the sustained sensitivity to TNFα induced necroptosis following fructose withdrawal. Significantly, siRNA mediated suppression of glut-5 expression prevented the fructose induced stimulation of both fructokinase and mitoNEET expression (Fig. 3A, lane #6). Also, prevention of fructokinase induction prevented the stimulation of mitoNEET expression, demonstrating that fructose metabolism is required to bring about induction of mitoNEET (Fig. 3A, lane #4). By contrast, suppression of mitoNEET expression had no effect on fructose induced stimulation of glut-5 or fructokinase expression, indicating that mitoNEET induction lies downstream of these two proteins (Fig. 3A, lane # 5). Also, pioglitazone did not prevent the induction of glut-5, fructokinase or mitoNEET expression by fructose, in keeping with its mechanism of binding to and disabling mitoNEET's ability to discharge its 2Fe-2S cluster (Fig. 3A, lane #8).

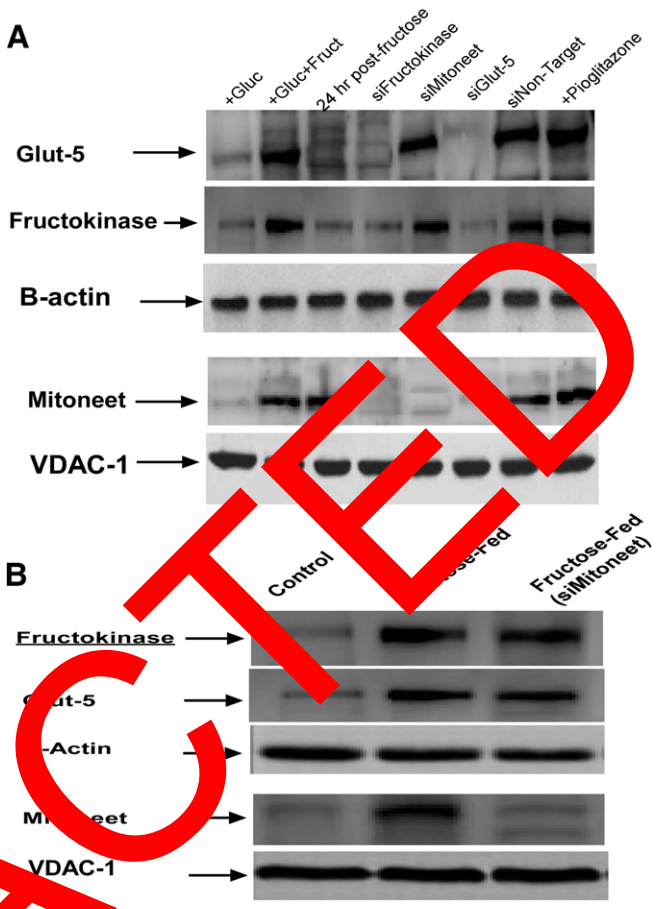

Fig. 3. Increased expression of glut-5, fructokinase and mitoNEET in pancreatic beta cells exposed to fructose and in beta cells isolated from fructose-fed mice. (A) INS-1 cells were plated in 6 well plates at 250,000 cells per well in basal RPMI media containing 5 mM of glucose. The cells were exposed to three 30 min spikes of 10 mM glucose alone or 1 mM fructose and 10 mM glucose over a 24 h period. Where indicated, following the last fructose-glucose spike, the cells were transfected with 50 nM of siRNA targeting mitoNEET, fructokinase, glut-5 or a non-targeting control siRNA and incubated for an additional 24 h. Alternatively, the cells were pre-treated with 10 μM pioglitazone. The cells were then harvested. Mitochondrial and cytosolic fractions were prepared and the level of mitoNEET, fructokinase and glut-5 were determined by western blotting. The results are typical of three independent experiments. (B) Pancreatic islets isolated from control or fructose-fed mice were incubated in basal RPMI media for 24 h. The islets of fructose-fed mice were then transfected with 50 nM of siRNA targeting mitoNEET or a non-targeting control siRNA and incubated for an additional 24 h. Approximately 200 islets were harvested, pooled and lysates prepared. Equal amounts of protein (30 μg) were resolved on 10% SDS-PAGE gels and transferred to PVDF membranes for western probing with antibodies against mitoNEET.

Importantly, islets isolated from fructose-fed mice also displayed elevations in the expression of mitoNEET, fructokinase and glut-5. Isolated pancreatic islets (200) were harvested, lysates prepared and utilized for western blotting. As shown in Fig. 3B, lane #1, pancreatic islets isolated from mice fed a control, non-fructose containing diet expressed low levels of glut-5, fructokinase or mitoNEET. However, feeding mice a diet with fructose constituting 20% of calories for two days resulted in a dramatic induction in the expression of glut-5, fructokinase and mitoNEET (Fig. 3B, lane #2). Importantly, the induction of mitoNEET expression was prevented by transfection of pancreatic islets with siRNA targeting mitoNEET (Fig. 3B, lane #3).

The induction of mitoNEET, fructokinase and glut-5 expression is required for fructose to sensitize beta cells to TNFα induced

RETRACTED

Biology Open (2015) 0, 1-11 doi:10.1242/bio.014712

necroptosis. INS-1E cells were transfected with siRNAs targeting glut-5, fructokinase, mitoNEET or a non-targeting control. Following 24 h, the cells were exposed to the three fructose-glucose spikes over 24 h. The cells were then treated with TNFα in basal media for the times indicated. As shown in Fig. 4A, suppression of glut-5, fructokinase or mitoNEET expression prevented TNFα and fructose induced cytotoxicity in INS-1E cells. Similarly, pancreatic islets were isolated from fructose-fed mice, dispersed and transfected with siRNAs. Following 24 h, the cells were then treated with TNFα in basal media for the times indicated. As show in Fig. 4B, suppression of glut-5, fructokinase or mitoNEET expression prevented TNFα induced ethidium monoazide staining of insulin positive islet cells. Additionally, INS-1E exposed to fructose or islets isolated from fructose-fed mice were pretreated with 1 μM of pioglitazone for 30 min prior to treatment with TNFα. As shown in Fig. 4A and B, pre-treatment with 1 μM pioglitazone prevented TNFα induced cytotoxicity in both INS-1E cells exposed to fructose and insulin positive islet cells isolated from fructose-fed mice, suggesting that the ability of pioglitazone to bind to and inhibit mitoNEET may account for its pro-survival effects (Paddock et al., 2007; Wiley et al., 2007b; Zuris et al., 2011). Importantly, non-target control siRNA had no effect on preventing TNFα induced cytotoxicity.

TNFα induced necroptosis is mediated by binding of the Stat3-Grim-19 complex to MitoNEET resulting in the release of its 2Fe-2S cluster, accumulation of mitochondrial iron and ROS production

INS-1E cells or pancreatic islets isolated from fructose fe[illegible]e were transfected with siRNAs targeting Grim-19, Stat3 or a [illegible]on-targeting control. For the INS-1E cells, following 24 h, the IN[illegible]1E cells were exposed to the three spikes of fructose-glucose. The media was then changed back to basal media and the cells were harvested. As shown in Fig. 5A, in INS-1 cells and islets, the siRNAs targeting Grim-19 or Stat3 selectively suppressed the expression of Grim-19 and Stat3, with the non-targeting control siRNA without effect on the expression of either protein. In Fig. 5B and C, the cells were treated with TNFα for the time periods indicated. Suppression of Stat3 or Grim-19 expression prevented TNFα induced cytotoxicity in fructose expos[illegible]S-1E cells (Fig. 5B). Similarly, as shown in Fig. 5C, in [illegible]sulin po[illegible]e islet cells isolated from fructose fed mice, su[illegible]ession of Gri[illegible]9 or Stat-3 expression also prevented TNFα in[illegible]ed cytotoxicity.

We have demonstrated that during TNFα [illegible]uced necropto[illegible]s of hepatocytes, Grim-19 and Stat-3 [illegible]nteract wit[illegible]e anoth[illegible] and translocate to the mitochond[illegible] (Shulga and Pa[illegible]rin[illegible] 2014). Therefore, INS-1E cells w[illegible] exposed [illegible]fructose-g[illegible]se spikes and then treated with TNFα. F[illegible]owin[illegible]wo hours, a time point prior to appreciable cell[illegible]eath, [illegible]ondria w[illegible] isolated and mitoNEET immun[illegible]ecipitated. A[illegible]how in[illegible]g. 6A, treatment of fructose expose[illegible]S-1E cells wi[illegible]α brought about an interaction be[illegible]een m[illegible]NEET and the Stat-3-Grim-19 complex that was prevented by ne[illegible]statin (lane 3# vs lane #4). Similarly, immun[illegible]itation of Grim[illegible]9 detected an interaction between it, Stat3 [illegible]nd mitoNEET in fructos[illegible]exposed cells treated with TNFα, wh[illegible]h was also prevented by necrostatin (Fig. 6B, bottom panels). [illegible]e have demons[illegible]ted that the binding of the Stat3-Grim-19 co[illegible]lex to mitoNE[illegible] initiates the release of mitoNEET's bound 2Fe-2[illegible]cluster. Mi[illegible]NEET's 2Fe-2S cluster can be monitored by its abso[illegible]t at 458 nm when the 2Fe-2S cluster is bound [illegible]ak et al., 2009; Zuris et al., 2011, 2012). MitoNEET was i[illegible]recipitated from isolated mitochondria and the

RETRACTED

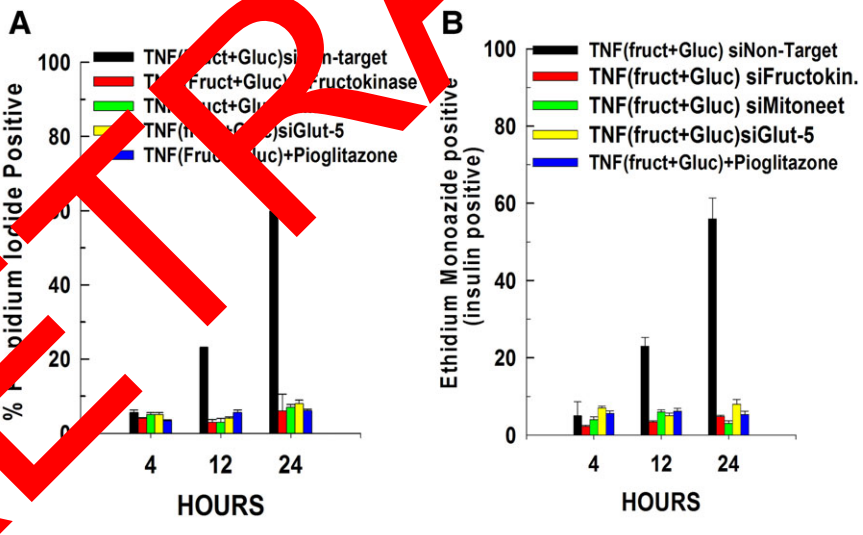

Fig. [illegible]pression of glut-5, fructokinase or mitoNEET expression or pretreatment with pioglitazone prevents TNFα induced necroptosis in pancre[illegible]beta cells. (A) INS-1E cells were plated at 50,000 cells per well in 24 well plates in 1 ml of basal RPMI media containing 5 mM of glucose. Following 24 h, the c[illegible] were exposed to three spikes of 1 mM fructose and 10 mM of glucose. Where indicated, after the last spike, the cells were transfected with 50 nM of siRNA targ[illegible]g mitoNEET, fructokinase, glut-5 or a non-targeting control siRNA and incubated for an additional 24 h. Alternatively, as indicated, the cells were pre-treated with 10 μM pioglitazone for 30 min following the last fructose-glucose spike. The cells were then treated with TNFα at 10 ng/ml for the time periods indicated. Following the treatment, the cells were harvested and viability assessed by propidium iodide uptake. Values are the means of three independent experiments with the error bars indicating s.d. (B) Mouse pancreatic islets were isolated from mice fed a non-fructose containing diet. The islets were dispersed, cultured for 24 h in basal RPMI media containing 5 mM of glucose. Over a 24 h period, the cells were exposed to three spikes of 1 mM fructose and 10 mM of glucose with an interval of eight hours between spikes. Where indicated, after the last spike, the cells were transfected with 50 nM of siRNA targeting mitoNEET, fructokinase, glut-5 or a non-targeting control siRNA and incubated for an additional 24 h. Alternatively, following the last fructose-glucose spike, the cells were pre-treated with 10 μM pioglitazone for 30 min. The cells were then treated with TNFα at 10 ng/ml for the time periods indicated. Cell viability was determined by the degree of ethidium monoazide staining of insulin positive cells. Values are the means of three independent experiments with the error bars indicating s.d.

Biology Open (2015) 0, 1-11 doi:10.1242/bio.014712

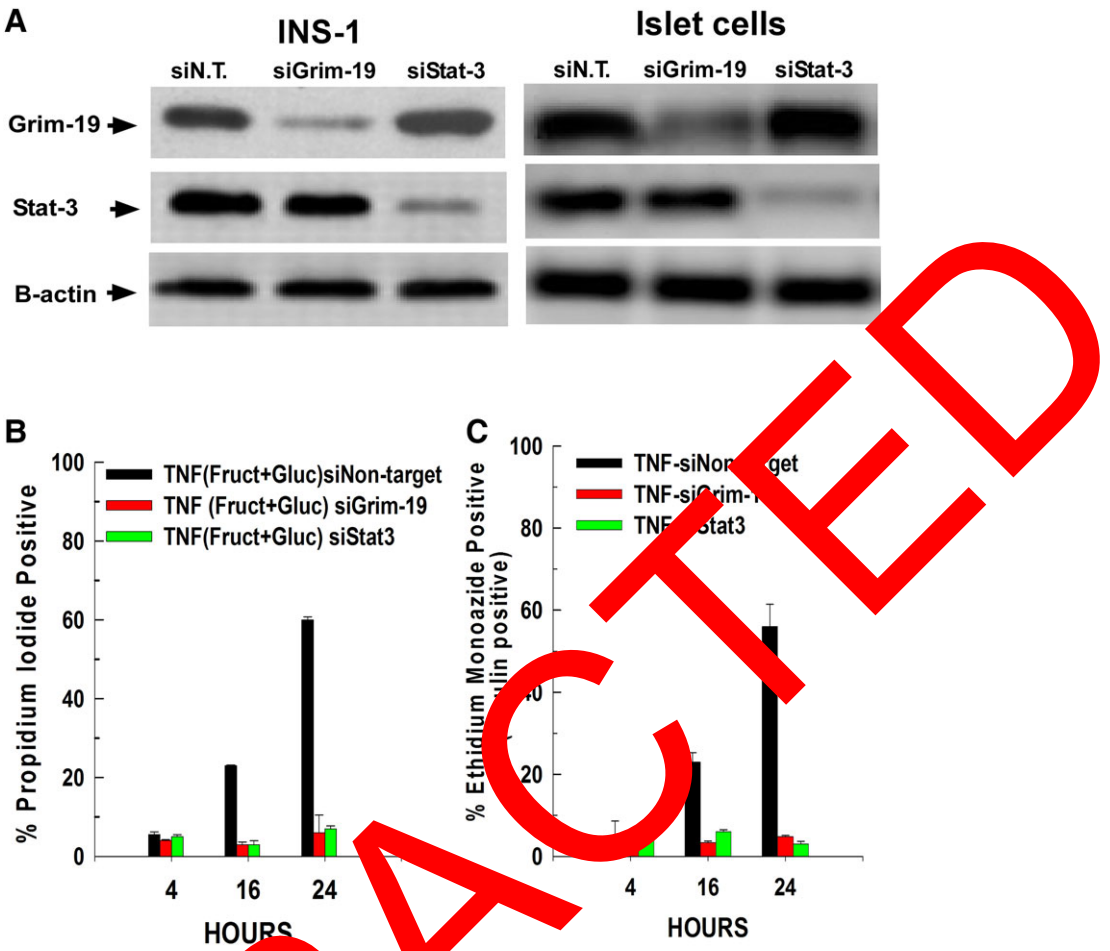

RETRACTED

Fig. 5. TNF induced necroptosis is dependent on Grim-19 and Stat3. (A) INS-1E cells were plated at 50,000 cells per well in 24 well plates in 1 ml of basal RPMI media containing 5 mM of glucose. Following 4 h, the cells were exposed to three spikes of 1 mM fructose and 10 mM of glucose. Where indicated, after the last spike, the cells were transfected with 50 nM of siRNA targeting grim-19, stat-3 or a non-targeting control siRNA and incubated for an additional 24 h. Alternatively, mouse pancreatic islets were isolated from mice fed a fructose containing diet. The islets were dispersed, cultured for 24 h in basal RPMI media containing 5 mM of glucose. Where indicated, the cells were transfected with 50 nM of siRNA targeting grim-19, stat-3 or a non-targeting control siRNA, and incubated for an additional 24 h. For INS-1E cells, the cells were then harvested. Cellular lysates were prepared and the levels of Grim-19, Stat3 or β-actin were determined by western blotting. These blots are typical of three independent experiments. For pancreatic islets, approximately 200 islets were harvested, pooled and lysates prepared. Equal amounts of protein (30 µg) were resolved on 10% SDS-PAGE gels and transferred to PVDF membranes for western probing with Grim-10, Stat3 or β-actin. (B) INS-1E cells were plated at 50,000 cells per well in 24 well plates in 1 ml of basal RPMI media containing 5 mM of glucose. Following 24 h, the cells were exposed to three spikes of 1 mM fructose and 10 mM of glucose. Where indicated, after the last spike, the cells were transfected with 50 nM of siRNA targeting grim-19, stat-3 or a non-targeting control siRNA and incubated for an additional 24 h. The cells were then treated with TNFα at 10 ng/ml for the time periods indicated. Following the treatment, the cells were harvested and viability assessed by propidium iodide uptake. Values are the means of three independent experiments with the error bars indicating s.d. (C) Mouse pancreatic islets were isolated from mice fed a fructose containing diet. The islets were dispersed, cultured for 24 h in basal RPMI media containing 5 mM of glucose. Where indicated, the cells were transfected with 50 nM of siRNA targeting grim-19, stat-3 or a non-targeting control siRNA, and incubated for an additional 24 h. The cells were then treated with TNFα at 10 ng/ml for the time periods indicated. Cell viability was determined by the degree of ethidium monoazide staining of insulin positive cells. Values are the means of three independent experiments with the error bars indicating s.d.

absorbance was measured. As show in Fig. 7A, treatment of fructose exposed INS-1E cells with TNFα induced a progressive decrease in absorbance at 458 nm, indicating a release of the 2Fe-2S cluster from mitoNEET that was not prevented by a non-targeting siRNA (green bars). The release of the 2Fe-2S cluster from mitoNEET is accompanied by an accumulation of mitochondrial iron; with the content of mitochondrial iron tripling within 4 h after treatment with TNFα in fructose exposed INS-1E cells (Fig. 7B, green bars). Moreover, suppression of Grim-19 expression or pre-treatment with pioglitazone prevented the discharge of the 2Fe-2S cluster from mitoNEET and accumulation of mitochondrial iron induced by TNFα in fructose exposed INS-1E cells (Fig. 7A and B). Suppression of mitoNEET expression also prevented the TNFα and fructose induced overload of mitochondrial iron (Fig. 7B).

The mitochondrial accumulation of iron resulted in a surge of ROS production. INS-1E cells exposed to fructose were treated with TNFα and mitochondrial ROS production was measured utilizing MitoSOX, which is somewhat selective at detecting ROS generated by mitochondria. As show in Fig. 8A, TNFα induced a robust stimulation of ROS production in fructose exposed INS-1E cells that was not prevented by a non-targeting siRNA (green bars). However, suppression of mitoNEET or Grim-19 expression prevented the TNFα induced stimulation of ROS production in fructose exposed cells. Also, pre-treatment of the fructose exposed INS-1E cells with pioglitazone prevented TNFα from stimulating

Biology Open (2015) 0, 1-11 doi:10.1242/bio.014712

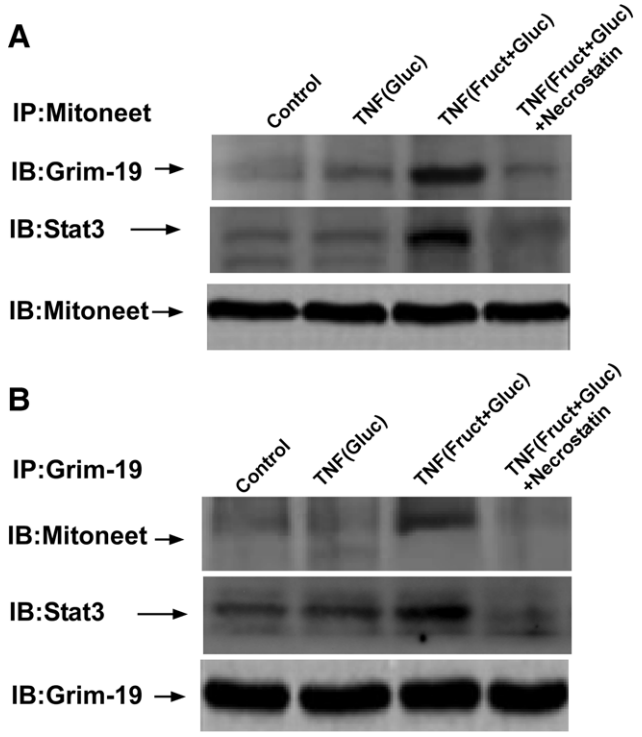

Fig. 6. TNFα induces binding of a Stat3-Grim-19 complex to mitoNEET in beta cells exposed to fructose. INS-1E cells were plated in 6 well plates at 250,000 cells per well in basal RPMI media containing 5 mM of glucose. The cells were exposed to three, 30 min spikes of 10 mM glucose alone or 5 mM fructose and 10 mM glucose over a 24 h period. Following the last spike the cells were treated with 10 ng/ml of TNFα. Alternatively, where indicated the cells were first pre-treated with 10 μM of necrostatin for 30 min before the addition of 10 ng/ml TNFα. After 4 h exposure to TNFα, the cells were harvested and mitochondria isolated. MitoNEET was immunoprecipitated from mitochondrial lysates. The immunoprecipitates were then separated by SDS-PAGE and blotted onto PVDF membranes. The blots were probed with antibodies against Grim-19 or Stat-3. For the reverse immunoprecipitation Grim-19 was immunoprecipitated from mitochondrial extracts and the immunoprecipitates separated by SDS-PAGE and blotted onto PVDF membranes. The blots were probed with antibodies against mitoNEET or Stat3. The results are representative of three independent experiments.

ROS production. Moreover, as shown in Fig. 8B, TNFα induced a similar surge of MitoSox fluorescence in the pancreatic beta cells of islets isolated from fructose fed mice that was prevented by pretreatment with pioglitazone, or suppression of Grim-19 or mitoNEET expression.

DISCUSSION

The effects of fructose on beta cell viability are not characterized. While the role of mitochondrial failure in beta cell injury and death is established, the signaling pathways and mechanisms that initiate mitochondrial injury are ill defined. The data define mitoNEET, a target of the anti-diabetic class of thiazolidinediones, as a distinct point of integration of mitochondrial dysfunction during TNFα induced cytotoxicity in fructose exposed pancreatic beta cells, relaying signals to the interior of the mitochondria, where ROS formation promotes opening of the mitochondrial permeability transition pore (PTP), resulting in mitochondrial depolarization and necrosis.

The reported serum concentration of fructose varies widely from 5 μM to 1.9 mM (Abdelmalek et al., 2012; Brymora et al., 2012; Kawasaki et al., 2012). It has been assumed that most fructose is metabolized in the liver and pancreatic beta cells possess little capacity to metabolize fructose. A study utilizing a fructose dehydrogenase assay in conjunction with GC-MS, found the fasting fructose serum concentration at 1.9 mM with a spike of 17.2 mM after ingestion of a fructose and glucose containing drink (Hui et al., 2009). Most studies do show a spike in serum fructose following its injection or ingestion. Importantly, our studies showed that mice fed 20% fructose in drinking water displayed a remarkable spike in the serum fructose concentration in just a short time frame, rising over four fold, from 1.8 mM to 8.4 mM.

Significantly, in our studies, for the 1 mM fructose spike to induce expression of glut5, fructokinase or mitoNEET in beta cells; it had to be accompanied by a spike in glucose (from 5 mM to 10 mM). This is analogous with findings that fructose absorption is increased in the presence of glucose in the intestine, a so called glucose dependent co-transport of fructose (Corpe et al., 1996; Le Gall et al., 2007; Wright et al., 2003). The main glucose transporter for the apical membrane of the enterocytes of the small intestine is glut-2, the same as in pancreatic beta cells. Therefore it is feasible that glucose assists the initial uptake of fructose into beta cells by glut-2. Once fructose is taken up, it subsequently induces the expression of glut-5, thereby enabling a greater uptake of fructose. Induction of fructokinase, with the resultant metabolic effects and subsequent induction of mitoNEET expression. Importantly, the induction of mitoNEET and fructokinase expression, in addition to the enhanced sensitivity incurred on pancreatic beta cells to TNFα induced cytotoxicity, was replicated in mice fed a fructose containing diet, indicating that in the intact pancreas, cells are exposed to fructose when it is a component of the diet.

We found that fructose exposure drastically lowers cellular ATP content by 50%. Unlike hexokinase or glucokinase; fructokinase does not experience feedback inhibition by fructose-1-phosphate, so unregulated fructokinase activity depletes ATP (Abdelmalek et al., 2012; Malaisse et al., 1989). Since mitoNEET is a 2Fe-2S transfer protein localized to the mitochondrial outer membrane, it is tempting to speculate that the up-regulation of mitoNEET expression is in response to fructose induced ATP depletion, resulting in an increased need for mitochondrial respiration that requires 2Fe-2S clusters in components of the mitochondrial respiratory chain. Intriguingly, a correlation has been identified between iron overload and the onset of diabetes, with reduction of iron though phlebotomy improving the deleterious effects of diabetes (Simcox and McClain, 2013; Tajima et al., 2012).

Exposure of pancreatic beta cells to either fructose or glucose has been demonstrated to promote onset of the mitochondrial permeability transition (Lablanche et al. 2011). In that work, fructose was found to be a more potent sensitizer to permeability transition pore opening than glucose. In this context it is important to note that opening of the permeability transition pore occurs during TNFα induced cytotoxicity (Pastorino et al., 1996).

In addition to the direct effects of fructose on the sensitivity of beta cells to TNFα induced cytotoxicity, fructose can affect the activation and function of innate inflammatory cells (Leibowitz et al., 2013). Fructose has been reported to induce endotoxemia in primates by providing an increase in the triggers necessary for promoting inflammatory cell activation (Kavanagh et al., 2013). Compounding this is the ability of fructose to increase the sensitivity of immune cells to activation (Tagzirt et al., 2014). These observations are consistent with studies demonstrating that the pancreatic islets in rat models of type II diabetes are infiltrated

RETRACTED

Biology Open • Advance article

Biology Open (2015) 0, 1-11 doi:10.1242/bio.014712

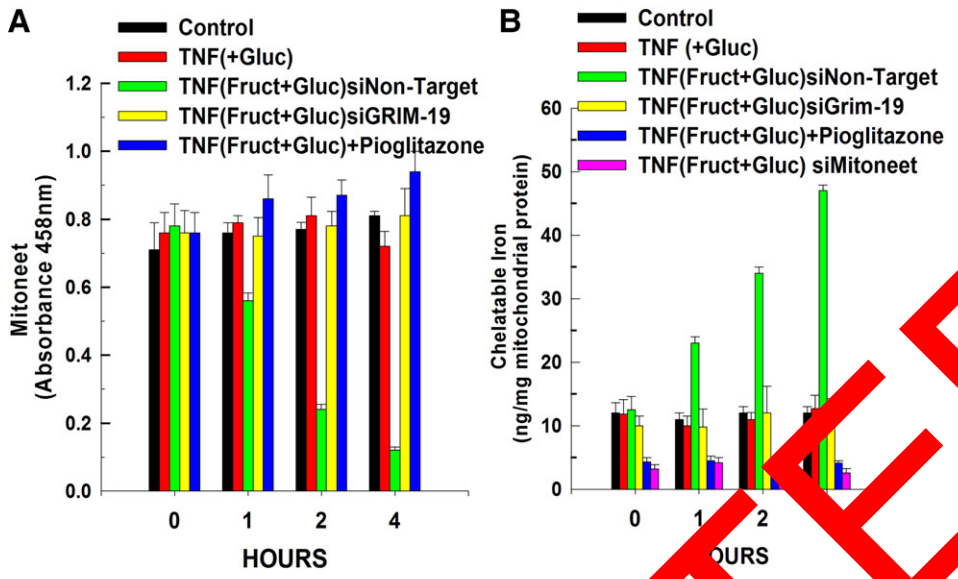

Fig. 7. TNFα induces release of the 2Fe-2S cluster bound to mitoNEET and accumulation of mitochondrial iron. (A) INS-1E cells were plated in 6 well plates at 250,000 cells per well in basal RPMI media containing 5 mM of glucose. Following 24 h, the cells were exposed to three spikes of 1 mM fructose and 10 mM of glucose at 8 h intervals. Where indicated, after the last spike, the cells were transfected with 50 nM of siRNA targeting grim-19, mitoNEET or a non-targeting control siRNA and incubated for an additional 24 h. Alternatively, the cells were pre-treated with 10 µM pioglitazone for 30 min. The cells were then treated with TNFα at 10 ng/ml. At the times indicated, the cells were harvested and mitochondria isolated. Mitochondrial lysates were prepared and mitoNEET immunoprecipitated. The absorbance of the immunoprecipitates was measured at 458 nm. Values are the means of three independent experiments with the error bars indicating s.d. (B) INS-1E cells were plated in 6 well plates at 250,000 cells per well in basal RPMI media containing 5 mM of glucose. The cells were exposed to three spikes of 1 mM fructose and 10 mM of glucose at 8 h intervals. Where indicated, after the last spike, the cells were transfected with 50 nM of siRNA targeting grim-19, mitoNEET or a non-targeting control siRNA and incubated an additional 24 h. Alternatively, the cells were pre-treated with 10 µM pioglitazone for 30 min. The cells were then treated with TNFα at 10 ng/ml. At the time periods indicated, the cells were harvested and mitochondria isolated. The content of mitochondrial iron was determined as described in Materials and Methods. Values are the means of three independent experiments with the error bars indicating s.d.

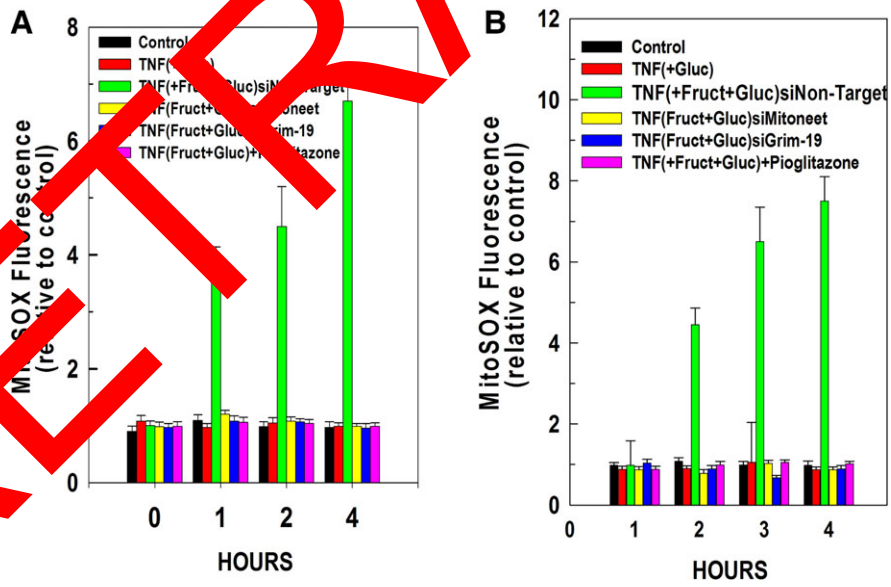

Fig. 8. Stimulation of ROS production in pancreatic beta cells exposed to fructose. (A) INS-1E cells were plated in 6 well plates at 250,000 cells per well in basal RPMI media containing 5 mM of glucose. At eight hour intervals, the cells were exposed to three spikes of 1 mM fructose and 10 mM of glucose. Where indicated, after the last spike, the cells were transfected with 50 nM of siRNA targeting grim-19, mitoNEET or a non-targeting control siRNA and incubated for an additional 24 h. Alternatively, the cells were pre-treated with 10 µM pioglitazone for 30 min. The cells were then treated with TNFα at 10 ng/ml. At the time points indicated, the cells were harvested and ROS formation was determined by measuring MitoSox fluorescence. Values are the means of three independent experiments with the error bars s.d. (B) Mouse pancreatic islets were isolated from mice fed a fructose containing diet. The islets were dispersed and cultured for 24 h in basal RPMI media containing 5 mM of glucose. Where indicated, the cells were then transfected with 50 nM of siRNA targeting grim-19, mitoNEET or a non-targeting control siRNA and incubated for an additional 24 h. Alternatively, the cells were pre-treated with 10 µM pioglitazone for 30 min. The cells were then treated with TNFα at 10 ng/ml. At the time points indicated, the cells were harvested and ROS formation was determined by measuring MitoSox fluorescence. Values are the means of three independent experiments with the error bars indicating s.d.

RETRACTED

Biology Open (2015) 0, 1-11 doi:10.1242/bio.014712

with activated macrophages (Cucak et al., 2014; Dahlen et al., 1998; Donath et al., 2013; Jourdan et al., 2013). All of these factors conspire to enhance the exposure of beta cells in pancreatic islets to damaging cytokines such as TNFα.

In summary, we find that fructose exposure induces the expression of fructokinase and mitoNEET in beta cells. Induction of mitoNEET expression was necessary for the sensitizing effect, as its suppression prevented the sensitization to TNFα induced cytotoxicity brought about by fructose exposure in the INS-1E and beta cells of pancreatic islets. In both the beta cells of pancreatic islets and INS-1E cells, the cytotoxicity induced by TNFα in the presence of fructose was a necroptotic form of cell killing. The data indicate that the induction of necroptosis in beta cells brought about by fructose exposure is partly caused by the TNFα induced binding of a Stat3-grim-19 complex to mitoNEET. Upon TNFα treatment, the binding of the Stat3-Grim-19 complex causes a discharge of mitoNEET's 2Fe-2S cluster, resulting in an accumulation of mitochondrial iron and ROS production. Suppression of either Stat3, Grim-19 or mitoNEET expression prevented the TNFα induced ROS production and necroptosis in beta cells exposed to fructose. Intriguingly, pre-treatment with the anti-diabetic drug, pioglitazone, which binds to and inhibits the ability of mitoNEET to release its 2Fe-2S cluster, prevented the TNFα induced accumulation of mitochondrial iron, ROS formation and cytotoxicity in fructose exposed pancreatic beta cells.

MATERIALS AND METHODS

Culture, isolation of pancreatic islets, and exposure of pancreatic beta cells to fructose spikes and TNFα

The pancreatic beta cells line, INS-1E, was cultured in basal RPMI containing 5 mM of glucose at 50,000 cells per well in 24 well plates for cell viability assays and at 250,000 cells per well in 6 well plates for harvesting and isolation of mitochondria. Where indicated, the cells were exposed to a 30 min spike of fructose (1 mM) and glucose (10 mM). In [illegible] cells were exposed to three, 30 min spikes of 1 mM fructose and 10 mM glucose over 24 h, with each spike separated by 8 h. Importantly, between the fructose-glucose spikes, the cells were washed with PBS and placed back into basal RPMI media prior to the next fructose-glucose spike. The cells were treated with TNFα at 10 ng/ml for the time periods indicated in the figures.

For isolation of pancreatic islets, eight to twelve week old male C57/BL6J mice are anesthetized with isoflurane. An abdominal incision was made to expose the liver and intestines. The ampulla is clamped with surgical clamps on the duodenum wall to block the bile duct pathway to the duodenum. Under a stereo-microscope, a 5 ml syringe with a 30G1/2-G needle is inserted into the common bile duct though the junction of the hepatic and cystic ducts, reaching the middle of the common bile duct. The pancreas is distended by injecting a solution of collagenase XI (1000 U/ml) in Hanks Balanced salt solution (HBSS). The pancreas is removed and placed in 50 ml of HBSS containing collagenase XI. The tube is incubated for 15 min at 37.5°C for digestion of the pancreas. The tube is shaken two or three times during the incubation. Following incubation, the pancreas is disrupted by shaking until a homogenous suspension is formed. The pancreatic digestion is stopped by placing the tube on ice and adding 25 ml of 1 mM $CaCl_2$ in HBSS. The tube is centrifuged at 300 ***g*** for 30 s at 4°C. The supernatant is decanted and the pellet resuspended in 25 ml of chilled HBSS containing 1 mM $CaCl_2$ wherein it is centrifuged for a second time at 300 ***g*** for 30 s. The supernatant is decanted and the pellet resuspended in 15 ml of HBSS containing 1 mM of $CaCl_2$. The resuspended pellet is poured over a 70 µm cell strainer. The contents of the strainer are deposited in a petri dish containing RPMI 1640 media. Under a stereo-microscope, islets are picked utilizing a wide mouthed pipette tip. The islets are placed into RPMI-1640 media and then into a 5% CO_2 incubator. The identity of islets cells was determined by immuno-staining for insulin (beta cells) or glucagon (alpha cells). Pancreatic islets were isolated from mice fed a non-fructose containing diet and from mice fed a fructose containing diet for two days. The mice were randomly divided into two groups, the control group and fructose-drinking water group. Both groups were fed a fructose-free rat chow diet. However the control group was given water while the fructose group was administered 20% fructose in the drinking water for two days.

Determination of fructose serum concentrations

The serum concentration of fructose was measured utilizing a spectrophotometric technique as described by Hui et al. (2009). A solution of fructose dehydrogenase from Gluconobacter sp (125 U/ml) was prepared in 1% Triton X-100, 1 mM 2-mercaptoethanol in citric phosphate buffer (0.05 M citric acid, 0.09 M dibasic phosphate, pH 4.5). MTT [3-(4,5-dimethylthiaze-syl)-2,5-diphenyltetrazolium bromide] was prepared in citric phosphate buffer at a concentration of 0.6 mg/ml with phenazine methosulfate (PMS) prepared at 0.4 mg/ml. Assays were performed in flat-bottom, 96-well microtiter plates. 20 µl of standard or serum was added to a well, and the assay initiated by the addition of 200 µl of mixing reagent. The plates were then incubated for 30 min at 37°C, followed by absorbance reading at 570 nm.

Determination of cell viability and ROS production

For INS-1E cells, viability was determined by the ability of the cells to exclude propidium iodide, an indicator of plasma membrane integrity. Following treatments, cells were harvested and centrifuged at 700 ×***g***. The cell pellet was re-suspended in phosphate buffered saline to which was added 5 µM of propidium iodide. After 5 min incubation, the cells were pelleted and re-suspended in PBS. The percentage of viable cells was determined utilizing a Cellometer (Nexcelom Bioscience LLC, Lawrence, MA, USA) as the ratio of the number of cells in the fluorescent images (propidium iodide positive) to the bright field images. To differentiate programmed, primary necrosis from secondary necrosis that is seen in late stages of apoptosis, annexin V staining was utilized concurrently. Cells undergoing programmed necrosis do not stain positive for annexin V at early time points and prior to loss of plasma membrane integrity, whereas apoptotic cells undergoing secondary necrosis will stain positive for annexin V.

Caspase activity was determined using NucView 488 Caspase-3 activity kit (Biotium Hayward, CA, USA). Floating and attached cells were collected and resuspended in RPMI media containing 5 µM of the NucView 488 substrate and then incubated at room temperature for 30 min protected from light. After incubation, the cells were washed once with ice cold PBS, and then re-suspended in PBS. Caspase activity was detected by an increase in the intensity of the DNA binding dye using Cellometer Vision (Nexcelom Bioscience). For determination of phosphatidylserine (PS) externalization, floating and attached cells were collected and re-suspended in 100 µl of binding buffer at 1.0×10^5 cells/ml. FITC-Annexin-V (5 µl) was added, and the cells were incubated for 15 min at room temperature. PS positive cells were determined by flow cytometry.

For isolated pancreatic islets, ethidium monoazide in conjunction with annexin V staining was utilized to determine the viability and mode of death for cells staining positive for insulin or glucagon. Ethidium monoazide is a fixable fluorescent photoaffinity label, which after photolysis, binds covalently to DNA and, like propidium iodide, only enters cells with compromised plasma membranes (Avlasevich et al., 2006).

As a positive control for apoptosis, beta cells were treated with staurosporine, a compound that brings about apoptosis in beta cells (Collier et al., 2011). Staurosporine induced caspase-3 activation along with annexin V staining before breakdown of plasma membrane integrity, as assessed by propidium iodide or ethidium monoazide.

For measurement of ROS production, 5 µM of MitoSOX was added to cells 10 min before harvesting. The cells were pelleted, re-suspended in PBS. In cells with active production of ROS, MitoSOX is oxidized to a fluorescent species. The percentage MitoSOX positive cells was determined utilizing a Cellometer which calculated the ratio of the number of MitoSOX positive cells in the fluorescence images to the number of cells in the bright field images.

Isolation of mitochondrial and cytosolic fraction

Following treatments, approximately 500,000 cells were harvested by trypsinization and centrifuged at 700×***g*** for 10 min at 4°C. The cell pellets were washed once in PBS and then resuspended in 3 volumes of isolation buffer (20 mM Hepes, pH 7.4, 10 mM KCl, 1.5 mM $MgCl_2$,

RETRACTED

Biology Open (2015) 0, 1-11 doi:10.1242/bio.014712

1 mM sodium EDTA, 1 mM dithiothreitol, and 10 mM phenylmethylsulfonyl fluoride, 10 µM leupeptin, 10 µM aprotinin) in 250 mM sucrose. After chilling on ice for 3 min, the cells were disrupted by 40 strokes of a glass homogenizer. The homogenate was centrifuged twice at 1500×***g*** at 4°C to remove unbroken cells and nuclei. The mitochondria-enriched fraction (heavy membrane fraction) was then pelleted by centrifugation at 12,000×***g*** for 30 min. The supernatant was removed and then filtered through a 0.2 µm and then a 0.1 µm Ultrafree-MC filter to obtain the cytosolic fraction.

Measurement of chelatable iron

Reagents were treated with Chelex 100 Resin (0.3 g/10 ml). The following reagents were incubated for 1 h at 37°C in a 1 ml final reaction volume: 100 mM Tris-HCl pH 7.4, 0.5 mg calf thymus DNA, 0.075 U bleomycin, 5 mM $MgCl_2$, 1 mM ascorbic acid, 50 µl of cell lysates or $FeCl_2$ standard. The reaction was stopped by the addition of 60 mM butylated hydroxytoluene. An aliquot of the mixture (0.4 ml) was incubated with 0.2 M phosphoric acid and 0.11 M thiobarbituric acid (TBA) for 45 min at 90°C. The upper organic layer was extracted with 1 ml N-butanol and absorbance was measured at 532 nm. A $FeCl_2$ concentration curve was constructed to determine the iron concentration in cellular lysates.

Transfection with siRNA

Cells or islets were transfected with the indicated siRNAs targeting mitoNEET, fructokinase, glut-5, Stat-3, Grim-19 or a non-targeting control utilizing a lipid-based method supplied from a commercial vendor (Gene Therapy Systems) at a final siRNA concentration of 50 nM. After formation of the siRNA-liposome complexes, the mixture was added to the cells for 8 h. Afterwards, the medium was aspirated, and complete medium was added back for a further 16 h, after which time the cells were utilized for experiments.

Immunoprecipitation of Grim-19 and Stat-3

Grim-19 or Stat-3 were immunoprecipitated from mitochondrial extracts (Grim-19 antibody from Novus Biologicals and Stat-3 antibody from Cell Signaling). The immunoprecipitates were then run out on SDS-PAGE gels and -blotted onto PVDF membranes. The western blots were developed with antibodies against Stat-3 or Grim-19.

Statistical analysis

Results are expressed as means±s.d. of at least three independent experiments. Statistical significance was defined at $P<0.05$.

Competing interests

The authors declare no competing or financial interests.

Author contributions

N.S. conducted experiments. J.G.P. conceived and performed experiments. J.G.P. prepared the manuscript.

Funding

This research received no specific grant from any funding agency in the public, commercial or not-for-profit sectors.

References

Abdul-Ghani, M. A., Puckett, C., Horska, A., Bonekamp, S., Lipkin, E. W., Balaraman, A., Bantle, J. P., Johnson, R. J., Diehl, A. M., Clark, J. M. et al. (2012). Higher dietary fructose is associated with impaired hepatic adenosine triphosphate homeostasis in obese individuals with type 2 diabetes. *Hepatology* **56**, 952-960.

Avlasevich, S. L., Bryce, S. M., Cairns, S. E. and Dertinger, S. D. (2006). In vitro micronucleus scoring by flow cytometry: differential staining of micronuclei versus apoptotic and necrotic chromatin enhances assay reliability. *Environ. Mol. Mutagen.* **47**, 56-66.

Bak, D. W., Zuris, J. A., Paddock, M. L., Jennings, P. A. and Elliott, S. J. (2009). Redox characterization of the FeS protein MitoNEET and impact of thiazolidinedione drug binding. *Biochemistry* **48**, 10193-10195.

Brymora, A., Flisinski, M., Johnson, R. J., Goszka, G., Stefanska, A. and Manitius, J. (2012). Low-fructose diet lowers blood pressure and inflammation in patients with chronic kidney disease. *Nephrol. Dial. Transplant.* **27**, 608-612.

Cnop, M., Welsh, N., Jonas, J.-C., Jorns, A., Lenzen, S. and Eizirik, D. L. (2005). Mechanisms of pancreatic beta-cell death in type 1 and type 2 diabetes: many differences, few similarities. *Diabetes* **54** Suppl. 2, S97-S107.

Collier, J. J., Burke, S. J., Eisenhauer, M. E., Lu, D., Sapp, R. C., Frydman, C. J. and Campagna, S. R. (2011) Pancreatic beta-cell death in response to pro-inflammatory cytokines is distinct from genuine apoptosis. *PLoS ONE* **6**, e22485.

Corpe, C. P., Basaleh, M. M., Affleck, J., Gould, G., Jess, T. J. and Kellett, G. L. (1996). The regulation of GLUT5 and GLUT2 activity in the adaptation of intestinal brush-border fructose transport in diabetes. *Pflugers Arch.* **432**, 192-201.

Cucak, H., Grunnet, L. G. and Rosendahl, A. (2014). Accumulation of M1-like macrophages in type 2 diabetic islets is followed by a systemic shift in macrophage polarization. *J. Leukoc. Biol.* **95**, 149-160.

Dahlen, E., Dawe, K., Ohlsson, L. and Hedlund, G. (1998). Dendritic cells and macrophages are the first and major producers of TNF-alpha in pancreatic islets in the nonobese diabetic mouse. *J. Immunol.* **160**, 3585-3593.

Dekker, M. J., Su, Q., Baker, C., Rutledge, A. C. and Adeli, K. (2010) Fructose: a highly lipogenic nutrient implicated in insulin resistance, hepatic steatosis, and the metabolic syndrome. *Am. J. Physiol. Endocrinol. Metab.* **299**, E685-E6[illegible]

Donath, M. Y., Dalmas, E., Sauter, N. S. and Boni-Schnetzler, M. (2013). Inflammation in obesity and diabetes: islet dysfunction and therapeutic opportunity. *Cell Metab.* **17**, 860-[illegible]

Douard, V. and Ferraris, R. P. (2008) Regulation of the fructose transporter GLUT5 in health and disease. *Am. J. Physiol. Endocrinol. Metab.* **295**, E227-E237.

Ehses, J. A., Boni-Schnetzler, M., Faulenbach, M. and Donath, M. Y. (2008). Macrophages, cytokines and beta-cell death in Type 2 diabetes. *Biochem. Soc. Trans.* **36**, 340-[illegible]

Goran, M. I., Ulijaszek, S. J. and Ventura, E. E. (2013) High fructose corn syrup and diabetes prevalence: a global perspective. *Glob. Public Health* **8**, 55-64.

Hui, H., Hu, D., McArthur, D., Nissen, N., Boros, L. G. and Heaney, A. P. (2009) Direct spectrophotometric determination of serum fructose in pancreatic cancer patients. *Pancreas* **38**, 706-712.

Ishimoto, T., Lanaspa, M. A., Le, M. T., Garcia, G. E., Diggle, C. P., MacLean, P. S., Jackman, M. R., Asipu, A., Roncal-Jimenez, C. A., Kosugi, T. et al. (2012). Opposing effects of fructokinase C and A isoforms on fructose-induced metabolic syndrome in mice. *Proc. Natl. Acad. Sci. USA* **109**, 4320-4325.

Jacobson, M. F. (2007) High-fructose corn syrup and the obesity epidemic. *Am. J. Clin. Nutr.* **85**, 1081; author reply 1081-1082.

Jurdan, T., Godlewski, G., Cinar, R., Bertola, A., Szanda, G., Liu, J., Tam, J., Mukhopadhyay, B., Skarulis, M. C. et al. (2013). Activation of the Nlrp3 inflammasome in infiltrating macrophages by endocannabinoids mediates beta cell loss in type 2 diabetes. *Nat. Med.* **19**, 1132-1140.

Kavanagh, K., Wylie, A. T., Tucker, K. L., Hamp, T. J., Gharaibeh, R. Z., Fodor, A. A. and Cullen, J. M. (2013). Dietary fructose induces endotoxemia and hepatic injury in calorically controlled primates. *Am. J. Clin. Nutr.* **98**, 349-357.

Kawasaki, T., Igarashi, K., Ogata, N., Oka, Y., Ichiyanagi, K. and Yamanouchi, T. (2012). Markedly increased serum and urinary fructose concentrations in diabetic patients with ketoacidosis or ketosis. *Acta Diabetol.* **49**, 119-123.

Kmietowicz, Z. (2012) Countries that use large amounts of high fructose corn syrup have higher rates of type 2 diabetes. *BMJ* **345**, e7994.

Koo, H.-Y., Wallig, M. A., Chung, B. H., Nara, T. Y., Cho, B. H. S. and Nakamura, M. T. (2008). Dietary fructose induces a wide range of genes with distinct shift in carbohydrate and lipid metabolism in fed and fasted rat liver. *Biochim. Biophys. Acta* **1782**, 341-348.

Krilanovich, N. J. (2004). Fructose misuse, the obesity epidemic, the special problems of the child, and a call to action. *Am. J. Clin. Nutr.* **80**, 1446-1447; author reply 1447-1448.

Lablanche, S., Cottet-Rousselle, C., Lamarche, F., Benhamou, P.-Y., Halimi, S., Leverve, X. and Fontaine, E. (2011) Protection of pancreatic INS-1 beta-cells from glucose- and fructose-induced cell death by inhibiting mitochondrial permeability transition with cyclosporin A or metformin. *Cell Death Dis.* **2**, e134.

Lanaspa, M. A., Sanchez-Lozada, L. G., Cicerchi, C., Li, N., Roncal-Jimenez, C. A., Ishimoto, T., Le, M., Garcia, G. E., Thomas, J. B., Rivard, C. J. et al. (2012). Uric acid stimulates fructokinase and accelerates fructose metabolism in the development of fatty liver. *PLoS ONE* **7**, e47948.

Lanaspa, M. A., Ishimoto, T., Cicerchi, C., Tamura, Y., Roncal-Jimenez, C. A., Chen, W., Tanabe, K., Andres-Hernando, A., Orlicky, D. J., Finol, E. et al. (2014). Endogenous fructose production and fructokinase activation mediate renal injury in diabetic nephropathy. *J. Am. Soc. Nephrol.* **25**, 2526-2538.

Le Gall, M., Tobin, V., Stolarczyk, E., Dalet, V., Leturque, A. and Brot-Laroche, E. (2007). Sugar sensing by enterocytes combines polarity, membrane bound detectors and sugar metabolism. *J. Cell. Physiol.* **213**, 834-843.

Leibowitz, A., Rehman, A., Paradis, P. and Schiffrin, E. L. (2013). Role of T regulatory lymphocytes in the pathogenesis of high-fructose diet-induced metabolic syndrome. *Hypertension* **61**, 1316-1321.

Lin, J., Zhou, T., Ye, K. and Wang, J. (2007). Crystal structure of human mitoNEET reveals distinct groups of iron sulfur proteins. *Proc. Natl. Acad. Sci. USA* **104**, 14640-14645.

Malaisse, W. J., Malaisse-Lagae, F., Davies, D. R. and Van Schaftingen, E. (1989). Presence of fructokinase in pancreatic islets. *FEBS Lett.* **255**, 175-178.

RETRACTED

Biology Open (2015) 0, 1-11 doi:10.1242/bio.014712

Medarova, Z., Bonner-Weir, S., Lipes, M. and Moore, A. (2005). Imaging beta-cell death with a near-infrared probe. *Diabetes* **54**, 1780-1788.

Mulder, H. and Ling, C. (2009). Mitochondrial dysfunction in pancreatic beta-cells in Type 2 diabetes. *Mol. Cell. Endocrinol.* **297**, 34-40.

Muoio, D. M. and Newgard, C. B. (2008). Mechanisms of disease: molecular and metabolic mechanisms of insulin resistance and beta-cell failure in type 2 diabetes. *Nat. Rev. Mol. Cell Biol.* **9**, 193-205.

Paddock, M. L., Wiley, S. E., Axelrod, H. L., Cohen, A. E., Roy, M., Abresch, E. C., Capraro, D., Murphy, A. N., Nechushtai, R., Dixon, J. E. et al. (2007). MitoNEET is a uniquely folded 2Fe 2S outer mitochondrial membrane protein stabilized by pioglitazone. *Proc. Natl. Acad. Sci. USA* **104**, 14342-14347.

Pastorino, J. G., Simbula, G., Yamamoto, K., Glascott, P. A., Jr, Rothman, R. J. and Farber, J. L. (1996). The cytotoxicity of tumor necrosis factor depends on induction of the mitochondrial permeability transition. *J. Biol. Chem.* **271**, 29792-29798.

Shulga, N. and Pastorino, J. G. (2012). GRIM-19-mediated translocation of STAT3 to mitochondria is necessary for TNF-induced necroptosis. *J. Cell Sci.* **125**, 2995-3003.

Shulga, N. and Pastorino, J. G. (2014a). Hexokinase II binding to mitochondria is necessary for Kupffer cell activation and is potentiated by ethanol exposure. *J. Biol. Chem.* **289**, 2613-2625.

Shulga, N. and Pastorino, J. G. (2014b). MitoNEET mediates TNFalpha-induced necroptosis promoted by exposure to fructose and ethanol. *J. Cell Sci.* **127**, 896-907.

Simcox, J. A. and McClain, D. A. (2013). Iron and diabetes risk. *Cell Metab.* **17**, 329-341.

Stephens, L. A., Thomas, H. E., Ming, L., Grell, M., Darwiche, R., Volodin, L. and Kay, T. W. (1999). Tumor necrosis factor-alpha-activated cell death pathways in NIT-1 insulinoma cells and primary pancreatic beta cells. *Endocrinology* **140**, 3219-3227.

Tagzirt, M., Corseaux, D., Pasquesoone, L., Mouquet, F., Roma-Lavisse, C., Ung, A., Lorenzi, R., Jude, B., Elkalioubie, A., Van Belle, E. et al. (2014). Alterations in neutrophil production and function at an early stage in the high-fructose rat model of metabolic syndrome. *Am. J. Hypertens.* **27**, 1096-1104.

Tajima, S., Ikeda, Y., Sawada, K., Yamano, N., Horinouchi, Y., Kihira, Y., Ishizawa, K., Izawa-Ishizawa, Y., Kawazoe, K., Tomita, S. et al. (2012). Iron reduction by deferoxamine leads to amelioration of adiposity via the regulation of oxidative stress and inflammation in obese and type 2 diabetes KKAy mice. *Am. J. Physiol. Endocrinol. Metab.* **302**, E77-E86.

Wagnerberger, S., Spruss, A., Kanuri, G., Volynets, V., Stahl, C., Bischoff, S. C. and Bergheim, I. (2012). Toll-like receptors 1-9 are el[illegible] in livers with fructose-induced hepatic steatosis. *Br. J. Nutr.* **107**, 17[illegible]738.

Wiley, S. E., Murphy, A. N., Ross, S. A., van der Geer, [illegible] and Dixon, J. [illegible]2007a). MitoNEET is an iron-containing outer mitoch[illegible]al membrane pro[illegible] that regulates oxidative capacity. *Proc. Natl. Acad. Sci[illegible]SA* **104**, 5318-5323.

Wiley, S. E., Paddock, M. L., Abresch, E[illegible]C., Gro[illegible]L., van der Ge[illegible] P., Nechushtai, R., Murphy, A. N., Jennin[illegible]P. A. and D[illegible]n, J. E. (200[illegible]. The outer mitochondrial membrane protei[illegible]itoNEET contains[illegible]ovel re[illegible]-active 2Fe-2S cluster. *J. Biol. Chem.* **282**[illegible]745-23749.

Wright, E. M., Martin, M. G. and T[illegible], E. (2003). I[illegible]stinal absorp[illegible]n health and disease–sugars. *Best Pract. Res[illegible]a. Gast[illegible]terol.* **17**, 943-956.

Zuris, J. A., Harir, Y., Con[illegible] A. R[illegible]y[illegible]man, M., Mi[illegible]eli, D., Tamir, S., Paddock, M. L., Onuch[illegible]J. N., Mittle[illegible]Cabantchi[illegible]I. et al. (2011) Facile transfer of [2Fe-2S][illegible]ters from the diab[illegible] drug t[illegible]get mitoNEET to an apo-acceptor protein. *[illegible]. Acad. Sci. USA*[illegible]047-13052.

Zuris, J. A., Ali, [illegible], Yeh, [illegible]guyen, T. A., Nec[illegible]shtai, R., Paddock, M. L. and Jennings, P. A. (2012) NA[illegible] inhibits [2Fe-2S] cluster protein transfer from diabetes drug target MitoNEE[illegible]an apo-acceptor protein. *J. Biol. Chem.* **287**, 1164[illegible]

RETRACTED
